# Heart rate variability as predictive factor for sudden cardiac death

**DOI:** 10.18632/aging.101386

**Published:** 2018-02-23

**Authors:** Francesco Sessa, Valenzano Anna, Giovanni Messina, Giuseppe Cibelli, Vincenzo Monda, Gabriella Marsala, Maria Ruberto, Antonio Biondi, Orazio Cascio, Giuseppe Bertozzi, Daniela Pisanelli, Francesca Maglietta, Antonietta Messina, Maria P. Mollica, Monica Salerno

**Affiliations:** 1 University of Foggia, Department of Clinical and Experimental Medicine, Foggia, Italy; 2 Università degli Studi di Napoli Federico II, Department of Experimental Medicine, Naples, Italy; 3 Struttura Complessa di Farmacia, Azienda Ospedaliero-Universitaria, Foggia, Italy; 4 CRD Center, Santa Maria del Pozzo, Somma Vesuviana (NA), Italy; 5 University of Catania, Department of Surgery, Catania, Italy; 6 University of Catania, Department of Anatomy, Catania, Italy; * Equal contribution

**Keywords:** heart rate, heart rate variability, sudden cardiac death, cardiovascular diseases, heart

## Abstract

Sudden cardiac death (SCD) represents about 25% of deaths in clinical cardiology. The identification of risk factors for SCD is the philosopher's stone of cardiology and the identification of non-invasive markers of risk of SCD remains one of the most important goals for the scientific community.

The aim of this review is to analyze the state of the art around the heart rate variability (HRV) as a predictor factor for SCD.

HRV is probably the most analyzed index in cardiovascular risk stratification technical literature, therefore an important number of models and methods have been developed.

Nowadays, low HRV has been shown to be independently predictive of increased mortality in post- myocardial infarction patients, heart failure patients, in contrast with the data of the general population.

Contrariwise, the relationship between HRV and SCD has received scarce attention in low-risk cohorts. Furthermore, in general population the attributable risk is modest and the cost/benefit ratio is not always convenient.

The HRV evaluation could become an important tool for health status in risks population, even though the use of HRV alone for risk stratification of SCD is limited and further studies are needed.

## Introduction

In the past decades, in high-income countries, thanks to the adoption of preventive measures, cardiovascular mortality was significantly reduced [1]. Nonetheless, cardiovascular diseases (CVD) are a leading cause of morbidity and mortality worldwide [2–5]. In developing countries, CVDs are responsible for approximately 17 million deaths every year. Sudden cardiac death (SCD) represent about 25% of these deaths, one of the most important and unresolved problems in clinical cardiology [6].

SCD in young people has an estimated incidence of 0.46-3.7 events per 100000 person-years and it is more frequent in men than women as summarized in Table 1 [8,9]. In young people it relates to channelopathies and cardiomyopathies [10–14], myocarditis and substance abuse [15–20]. The risk of SCD increases with age due to the higher prevalence of coronary artery disease (CAD), valvular heart diseases and heart failure (HF) in older age [7]. To ascertain the cause of the death in young people, the autopsy remains the main procedure, along with histological, immunohistochemical, toxicological, and genetic examinations; nonetheless, about 48% of these deaths result unexplained [10].

**Table 1:** SCD rate estimated per 100000 person-years: the risk of SCD is higher in men than in women [7].

	**MEN**	**WOMEN**
**SCD rate estimated per 100000 person-years**	6.68 (95% CI - 6.24, 7.14)	1,46 (95% CI - 0.95, 1.98)

The identification of risk factors for SCD is the philosopher's stone of cardiology. In the major number of cases (>90%), the victim has previously known or unrecognized cardiac abnormality [21,22]. To date, this field of research is full of difficulties because the phenomenon of SCD originates as a ‘perfect storm’, interacting with a vulnerable substrate (genetic or acquired changes in the electrical or mechanical properties of the heart) with multiple transient factors that participate in triggering the fatal event [23].

Several studies have provided evidence that the predisposition to die suddenly can be traced in the genes, even in absence of a Mendelian disease, and encourages molecular investigations to identify DNA markers to predict SCD in the general population [24,25].

In the last decades, researchers throughout the world tried to identify a broad range of ‘markers’ for SCD. Besides utilizing public access defibrillation (PAD) procedure to rescue impending death patient after collapse, the better way is to prevent onset SCD by adopting medical aid prior to collapse [26]. For patients with myocardial ischemia, more "indicators" have been proposed, such as programmed ventricular stimulation (PVS), late potentials, heart rate variability (HRV), baroreflex sensitivity, QT interval dispersion, microvolt T-wave alternans and heart rate turbulence [27].

Among these, HRV proved to be the most interesting marker. It is considered, indeed, a standard noninvasive method for evaluating Autonomic Nervous System (ANS) function [28]. Previous studies have shown that HRV is a powerful prognostic indicator of arrhythmic events following myocardial infarction [29,30]. Sympathetic activity is associated with the low frequency range (LF, 0.04–0.15 Hz) while parasympathetic activity is associated with the higher frequency range (HF, 0.15–0.4 Hz) of modulation frequencies of the heart rate [31]. This frequency range difference represents an important marker to identify the contribution of sympathetic and parasympathetic systems. As well described in literature, lower HRV is frequently related to a poorer autonomic function [32,33], and this parameter is the most extensively studied among all arrhythmia risk markers.

## ECG

The detection of electrocardiographic (ECG) and echocardiographic signs for the identification of SCD represents an essential part of clinical practice and it is at the base of the identification of patients at risk of SCD. In ECG continuous, each QRS complex is recorded: all intervals between adjacent QRS complexes, resulting from sinus node depolarization, named normal to normal (NN) intervals, determined the instantaneous hart rate.

An important question for the scientific community remains if these techniques must be used as mass screening in populations at risk of sudden death. For example, Italy and Japan have implemented ECG screening systems, with the aim to identify asymptomatic patients with inheritable arrhythmogenic diseases [34–36]. The evaluation of the cost/benefit profile of performing ECG screening in different populations and in different healthcare systems and settings should be carried out. Notwithstanding the recent studies, there is no clear data supporting the benefit of broad screening programs in the general population [37,38]. Nonetheless, for what concerns the inclusion of ECG as an essential technique for the prevention of SCD in adolescent athletes, the European Society of Cardiology suggests the incorporation [39,40], while the American Heart Association considers more important personal/family history and physical examination [41].

However, a standard resting 12-lead ECG can identify the principal inherited disorders associated with ventricular arrhythmias (VAs) and SCD such as channelopathies (Long Q-T Syndrome, Short Q-T Syndrome, Brugada syndrome, Catecholaminergic polymorphic ventricular tachycardia) and cardiomyopathies (Arrhythmogenic right ventricular dysplasia and Hypertrophic cardiomyopathy). Furthermore, the ECG techniques can contribute identifying structural disease including bundle branch block, atrioventricular (AV) block, ventricular hypertrophy and Q waves consistent with ischemic heart disease or infiltrative cardiomyopathy. Electrolyte disturbances and the effects of various drugs may result in the repolarization of abnormalities and/or in the prolongation of the QRS duration. Exercise stress testing combined with ECG testing has been used for the detection of silent ischemia in adult patients with VAs. Exercise-induced non-sustained ventricular tachycardia (V-tach or VT) was reported in nearly 4% of asymptomatic middle-aged adults and was not associated with an increased risk of total mortality [42].

## Heart rate (HR) and HR variability (HRV)

HR and HRV provide a measure of how the organism reacts and adapts itself to stress, physical fatigue and metabolic-request changes [43].

The intrinsic HR generated by the sinoatrial node (SA node) in the absence of any neural or hormonal influence is about 100 to 120 beats per minute (BPM). However, in healthy individual, resting HR would never be that high. The normal resting adult human heart rate ranges from 70–75 bpm, but this value decreased in trained people (around 60 bpm). The exercise training influences the parasympathetic tone, reducing HR [44]. In fact, practicing endurance sport can lead to slow heart rate below 60 bpm at rest, so-called "bradycardia", and it is considered a training status index [45]. Several studies, on the other hand, indicate that the normal resting adult heart rate is probably closer to a range between 50 and 90 bpm. During sleep a slow heartbeat with rates around 40–50 bpm is common and is considered normal. When the heart is not beating in a regular pattern, this is referred to as an arrhythmia [46,47].

The period of time between two consecutive beats is called cardiac period (CP); the duration time is not the same generating the HRV, defined as the fluctuation degree of HR around its mean value [48]. HRV is mirroring the regularity of heart beats: bigger regularity - lowers HRV (and vice versa). Regularity of heartbeats is derived from a quantity of values; equal to the times elapsed between successive heartbeats. They are named R-R intervals and are measured in millisecond (ms). R-R intervals are obtained from ECG or tachograph. The physiological origins of HRV are the fluctuations of the activity of cardiovascular vasoconstrictor and vasodilator centres in brain. Normally these fluctuations are a result of blood pressure oscillation (baroreflex modulated), respiration, thermoregulation, and circadian biorhythm. All these factors can influence the length of beat-to-beat intervals, named R-R intervals.

Politano et al. [49] described different methods to determine HRV: time domain methods, frequency domain methods, short-term recording and long-term recording. Analyzing the differences among HRV measures, the SDNN (Standard Deviation of Normal-to-Normal R-R intervals) is not a well statistically method because it is related to the recording period. The SDANN (Standard Deviation of the 5-minute Average NN intervals) represents an estimate of the heart changes in two periods with a duration of 5 minutes. Frequently, the measure of the root-mean-square of the difference of successive R-R intervals (RMSSD) was used. As a frequency domain method, the power spectral density (PSD) analyzes the relationship between power and frequency. In the category of short-term recording (2-5 minutes) Very Low Frequency (VLF), Low Frequency (LF) and High Frequency (HF) are analyzed. In the category of long-term recordings, the same parameters are evaluated in the 24-hour period (Table 2).

**Table 2:** Parameters utilized for HRV analysis [49].

**MEASUREMENT OF HRV**		**SPECTRAL COMPONENTS**	
**TIME DOMAIN METHODS**	**FREQUENCY DOMAIN METHODS**	**SHORT TERM RECORDING** **(2-5 minutes)**	**LONG-TERM RECORDING** **(24 hour)**
**Standard deviation of the NN intervals (SDNN)**	Power spectral density (PSD)	Very Low Frequency (VLF) (Milliseconds squared)	Very Low Frequency (VLF)
**Standard deviation of the average NN intervals (SDANN)**	PSD parametric	Low Frequency (LF) (Milliseconds squared)	Low Frequency (LF)
**Root mean square of difference of successive RR intervals (RMSSD)**	PSD non parametric	High Frequency (HF) (Milliseconds squared)	High Frequency (HF)

**Figure 1:**
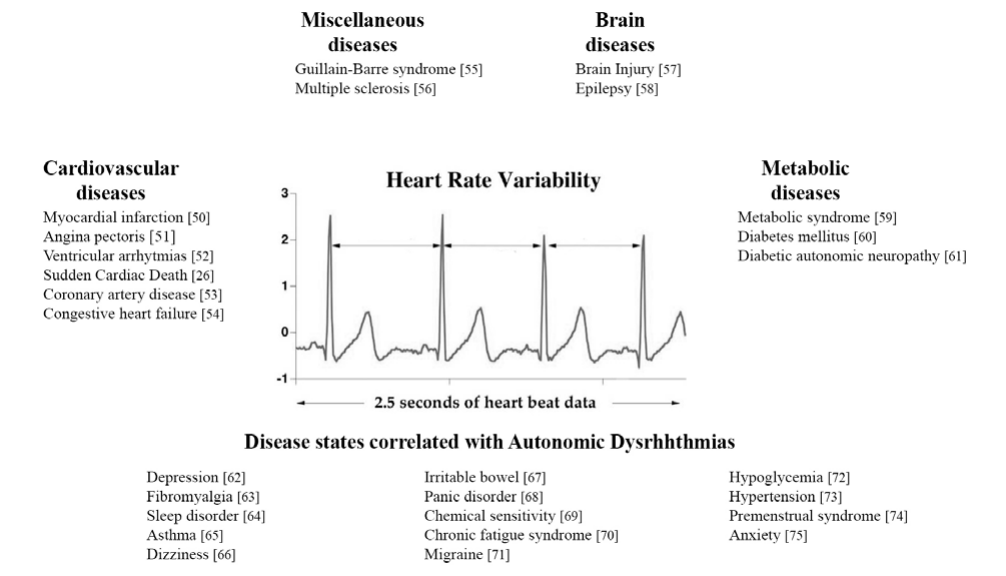
Principal diseases related to HRV.

The HRV is related to a wide broad spectrum of disease or symptoms [26,50–75], as illustrated in Figure 1.

The clinical use of HRV was described for the first time in 1963 [76], but it became a strong and important factor in the death cases after heart failure during the 90s [77–79]. Thanks to studies on animal models the true nature of HRV was discovered: the lower frequencies of the power spectrum of HRV are under control of the sympathetic nervous system directed to the heart, whereas higher frequencies are regulated by vagal activity modulated by respiration and depict respiratory sine arrhythmia [80,81]. On the other hand, thanks to HRV power spectral analysis, several studies described that a lower respiratory sinus arrhythmia connected with deep breathing tests are related to cardiac vagal dysfunction in obese adolescents [28]. Moreover, the HRV marker was frequently investigated in women with the aim to identify the relationship among obesity (analyzing lean and obese groups) and menopausal age (premenopausal and postmenopausal group). The power spectral analysis of HRV highlighted a significant reduction in LF and HF both in obesity group and in postmenopausal group. These findings are related to a reduction of sympathetic and parasympathetic activities: the reduction of the sympathetic branch is directly linked to obesity [31]. On the other hand, a reduction of the parasympathetic activity is associated with different conditions related to higher rate of morbidity and mortality [28].

In elderly individuals, the HR spectrum was observed lower because respiratory sine arrhythmia is lost with ageing [82]. In diseased individuals with heart failure, reduced or abnormal HRV are indicators of an increased risk of mortality [83–85]. HRV has proved to be useful in predicting SCD in patients suffering from non-cardiac causes [86,87].

To date, even though both HR and HRV are related with cardiovascular risk and SCD, not always they have been used to evaluate the health conditions of athletes [88–92].

It is very interesting that athletes appear at excessive risk of SCD compared with similar-aged non-athletes [93]; the annual incidence of SCD in young athletes (<35 years) is estimated to range from 0.7 to 3.0 per 100 000 athletes [94]. In older athletes, the incidence is higher and is expected to increase with age [95]. The intensity of the activity and the age of the athlete are core risk factors.

It is well known that an intense training forces the entire body to adapt to this condition through the development of morphological and functional changes [96–98]. These adjustments mean that the heart of an athlete appears different from that a sedentary subject [99]. Typically, SCD occurs in athletes when pathological hypertrophic enlargement of the heart went undetected or it was incorrectly attributed to benign athletic adaptations. Other alternative causes of SCD are: episodes of isolated arrhythmias which degenerated into lethal ventricular fibrillation and asystole, and undetected asymptomatic congenital defects of the cardiac valves. For these reasons, coaches and staff at sporting facilities are trained to face emergency situations, perform cardiopulmonary resuscitation and use automatic external defibrillators [100,101].

In 2001, Lombardi et al. examined the role of HRV for identifying patients at risk of SCD, concluding that the measurement of HRV represents a useful research tool for documenting events in various clinical settings, even if the specificity and predictive accuracy of imminent or future fatal arrhythmia events have been relatively low yet. Another study [102] suggests that short-term HRV recordings should be routine in chronic heart failure (CHF) patients. In CHF, markedly reduced HRV has been demonstrated to coincide with the severity of CHF, as well as being an independent marker of sympatho-excitation. On the other hand, after acute myocardial infarction, depressed HRV predicts cardiac mortality and malignant arrhythmias [103,104]. In 2007 Kiviniemi et al [105]. demonstrated that HRV spectral parameters (Table 2), had a strong prognostic power as predictor factors of SCD.

Ebrahimzadeh et al. [26] stated that the four-minute interval before SCD contains more information related to the SCD which can be used for prediction. They further specified that the first one-minute interval before SCD contains much more precious information for prediction of SCD in comparison with other intervals.

Finally, Maranesi et al. [43] described an innovative low-cost, large-scale procedure for HR and HRV monitoring from signals obtained using comfortable wearable sensors in order to evaluate the health status of an athlete besides his/her performance levels. Thanks to tachograph, they have monitored 10 amateur athletes in contrast with 10 sedentary subjects, with no differences in term of age, weight and height. They acquired tachogram at resting phase, during exercise and in the recovery phase. Their results suggest that physical activity has a beneficial effect on health status (RMSSD lower than normal, HR lower and HRV higher at resting phase).

## Discussion and conclusion

The SNA controls every aspect of cardiac activity. It is well described that autonomic instability and CNS disorders can lead to cardiovascular diseases. As previously analyzed, the autonomic cardiac innervations are involved in SCD. Their role is important in cardiac excitability and propagation [106].

HRV is probably the most analyzed index in the cardiovascular risk stratification technical literature, and an important number of models and methods have been developed for this purpose. As previously described, the HRV represent an accessible technique for evaluating the risks of SCD. There are several open questions for the scientific community related to use of HR and HRV as predictor factors of SCD, such as economic feasibility, applicability in mass screening and comfort of the measurements.

Nowadays, low HRV has been shown to be independently predictive of increased mortality in post- myocardial infarction patients, CHF patients, in contrast with the data of the general population.

Contrariwise, the relationship between HRV and SCD has received limited evaluation in low-risk cohorts. Furthermore, in general population the attributable risk is modest and the cost/benefit ratio is not always convenient. The HRV evaluation could be an important tool for health status in risks population, such as athletes, subject with familiarity, etc. For example, SCD remains the leading medical cause of death in athletes, even if, the precise causes are unclear. To date, the prevention of the SCD represents a broad ethical challenge, as it requires balancing the benefits and risks of an inappropriate decision for an athlete [107].

In this scenario, further studies are required to determine if the inclusion of HRV in a multi-marker approach would improve risk prediction of SCD in the general population. To date, the use of HRV alone for risk stratification of SCD is limited.

## References

[1] Niemeijer MN, van den Berg ME, Leening MJ, Hofman A, Franco OH, Deckers JW, Heeringa J, Rijnbeek PR, Stricker BH, Eijgelsheim M. Declining incidence of sudden cardiac death from 1990-2010 in a general middle-aged and elderly population: the Rotterdam Study. Heart Rhythm. 2015; 12:123–29. 10.1016/j.hrthm.2014.09.054 25277989

[2] Benjamin EJ, Blaha MJ, Chiuve SE, Cushman M, Das SR, Deo R, de Ferranti SD, Floyd J, Fornage M, Gillespie C, Isasi CR, Jimenez MC, Jordan LC, et al, and American Heart Association Statistics Committee and Stroke Statistics Subcommittee. Heart disease and stroke statistics—2017 update: a report from the American Heart Association. Circulation. 2017; 136:e196. 10.1161/CIR.0000000000000530 28122885 PMC5408160

[3] Kikuchi K, Poss KD. Cardiac regenerative capacity and mechanisms. Annu Rev Cell Dev Biol. 2012; 28:719–41. 10.1146/annurev-cellbio-101011-155739 23057748 PMC3586268

[4] Oh H, Bradfute SB, Gallardo TD, Nakamura T, Gaussin V, Mishina Y, Pocius J, Michael LH, Behringer RR, Garry DJ, Entman ML, Schneider MD. Cardiac progenitor cells from adult myocardium: homing, differentiation, and fusion after infarction. Proc Natl Acad Sci USA. 2003; 100:12313–18. 10.1073/pnas.2132126100 14530411 PMC218755

[5] Neri M, Fineschi V, Di Paolo M, Pomara C, Riezzo I, Turillazzi E, Cerretani D. Cardiac oxidative stress and inflammatory cytokines response after myocardial infarction. Curr Vasc Pharmacol. 2015; 13:26–36. 10.2174/15701611113119990003 23628007

[6] Moran AE, Roth GA, Narula J, Mensah GA. 1990-2010 global cardiovascular disease atlas. Glob Heart. 2014; 9:3–16. 10.1016/j.gheart.2014.03.1220 25432106

[7] Eckart RE, Shry EA, Burke AP, McNear JA, Appel DA, Castillo-Rojas LM, Avedissian L, Pearse LA, Potter RN, Tremaine L, Gentlesk PJ, Huffer L, Reich SS, Stevenson WG, and Department of Defense Cardiovascular Death Registry Group. Sudden death in young adults: an autopsy-based series of a population undergoing active surveillance. J Am Coll Cardiol. 2011; 58:1254–61. 10.1016/j.jacc.2011.01.049 21903060

[8] Maron BJ, Gohman TE, Aeppli D. Prevalence of sudden cardiac death during competitive sports activities in Minnesota high school athletes. J Am Coll Cardiol. 1998; 32:1881–84. 10.1016/S0735-1097(98)00491-4 9857867

[9] van der Werf C, Hendrix A, Birnie E, Bots ML, Vink A, Bardai A, Blom MT, Bosch J, Bruins W, Das CK, Koster RW, Naujocks T, Schaap B, et al. Improving usual care after sudden death in the young with focus on inherited cardiac diseases (the CAREFUL study): a community-based intervention study. Europace. 2016; 18:592–601. 10.1093/europace/euv059 25833117

[10] Mazzanti A, O’Rourke S, Ng K, Miceli C, Borio G, Curcio A, Esposito F, Napolitano C, Priori SG. The usual suspects in sudden cardiac death of the young: a focus on inherited arrhythmogenic diseases. Expert Rev Cardiovasc Ther. 2014; 12:499–519. 10.1586/14779072.2014.894884 24650315

[11] Bafunno V, Bury L, Tiscia GL, Fierro T, Favuzzi G, Caliandro R, Sessa F, Grandone E, Margaglione M, Gresele P. A novel congenital dysprothrombinemia leading to defective prothrombin maturation. Thromb Res. 2014; 134:1135–41. 10.1016/j.thromres.2014.08.028 25242243

[12] Pilmer CM, Kirsh JA, Hildebrandt D, Krahn AD, Gow RM. Sudden cardiac death in children and adolescents between 1 and 19 years of age. Heart Rhythm. 2014; 11:239–45. 10.1016/j.hrthm.2013.11.006 24239636

[13] Pellegrino PL, Bafunno V, Ieva R, Brunetti ND, Mavilio G, Sessa F, Grimaldi M, Margaglione M, Di Biase M. A novel mutation in human ether-a-go-go-related gene, alanine to proline at position 490, found in a large family with autosomal dominant long QT syndrome. Am J Cardiol. 2007; 99:1737–40. 10.1016/j.amjcard.2007.01.056 17560885

[14] Asatryan B, Vital C, Kellerhals C, Medeiros-Domingo A, Gräni C, Trachsel LD, Schmied CM, Saguner AM, Eser P, Herzig D, Bolliger S, Michaud K, Wilhelm M. Sports-related sudden cardiac deaths in the young population of Switzerland. PLoS One. 2017; 12:e0174434. 10.1371/journal.pone.0174434 28350812 PMC5370100

[15] McLain LG. Sudden death in young athletes. Pediatr Ann. 2003; 32:720–24, 724. 10.3928/0090-4481-20031101-04 22111152

[16] Turillazzi E, Bello S, Neri M, Pomara C, Riezzo I, Fineschi V. Cardiovascular effects of cocaine: cellular, ionic and molecular mechanisms. Curr Med Chem. 2012; 19:5664–76. 10.2174/092986712803988848 22856657

[17] Landry CH, Allan KS, Connelly KA, Cunningham K, Morrison LJ, Dorian P, and Rescu Investigators. Sudden cardiac arrest during participation in competitive sports. N Engl J Med. 2017; 377:1943–53. 10.1056/NEJMoa1615710 29141175 PMC5726886

[18] Pomara C, Bello S, D’Errico S, Greco M, Fineschi V. Sudden death due to a dissecting intramural hematoma of the esophagus (DIHE) in a woman with severe neurofibromatosis-related scoliosis. Forensic Sci Int. 2013; 228:e71–75. 10.1016/j.forsciint.2013.02.005 23453641

[19] Piacentino D, Kotzalidis GD, Del Casale A, Aromatario MR, Pomara C, Girardi P, Sani G. Anabolic-androgenic steroid use and psychopathology in athletes. A systematic review. Curr Neuropharmacol. 2015; 13:101–21. 10.2174/1570159X13666141210222725 26074746 PMC4462035

[20] Zhao Y, Feng Y, Ding X, Dong S, Zhang H, Ding J, Xia X. Identification of a novel hypertrophic cardiomyopathy-associated mutation using targeted next-generation sequencing. Int J Mol Med. 2017; 40:121–29. 10.3892/ijmm.2017.2986 28498465 PMC5466385

[21] Wever EF, Hauer RN, Oomen A, Peters RH, Bakker PF, Robles de Medina EO. Unfavorable outcome in patients with primary electrical disease who survived an episode of ventricular fibrillation. Circulation. 1993; 88:1021–29. 10.1161/01.CIR.88.3.1021 8353864

[22] Chugh SS. Sudden cardiac death with apparently normal heart: clinical implications of progress in pathophysiology. Card Electrophysiol Rev. 2001; 5:394–402. 10.1023/A:1013254132689

[23] Wellens HJ, Schwartz PJ, Lindemans FW, Buxton AE, Goldberger JJ, Hohnloser SH, Huikuri HV, Kääb S, La Rovere MT, Malik M, Myerburg RJ, Simoons ML, Swedberg K, et al. Risk stratification for sudden cardiac death: current status and challenges for the future. Eur Heart J. 2014; 35:1642–51. 10.1093/eurheartj/ehu176 24801071 PMC4076664

[24] Norrish G, Cantarutti N, Pissaridou E, Ridout DA, Limongelli G, Elliott PM, Kaski JP. Risk factors for sudden cardiac death in childhood hypertrophic cardiomyopathy: A systematic review and meta-analysis. Eur J Prev Cardiol. 2017; 24:1220–30. 10.1177/2047487317702519 28482693

[25] Calcagnino M, Crocamo A, Ardissino D. Genetic testing in predicting the risk of sudden death. J Cardiovasc Med (Hagerstown). 2017 (Suppl 1); 18:e64–66. 10.2459/JCM.0000000000000477 28009642

[26] Ebrahimzadeh E, Pooyan M, Bijar A. A novel approach to predict sudden cardiac death (SCD) using nonlinear and time-frequency analyses from HRV signals. PLoS One. 2014; 9:e81896. 10.1371/journal.pone.0081896 24504331 PMC3913584

[27] Gimeno-Blanes FJ, Blanco-Velasco M, Barquero-Pérez Ó, García-Alberola A, Rojo-Álvarez JL. Sudden cardiac risk stratification with electrocardiographic indices - A review on computational processing, technology transfer, and scientific evidence. Front Physiol. 2016; 7:82. 10.3389/fphys.2016.00082 27014083 PMC4780431

[28] Messina G, Vicidomini C, Viggiano A, Tafuri D, Cozza V, Cibelli G, Devastato A, De Luca B, Monda M. Enhanced parasympathetic activity of sportive women is paradoxically associated to enhanced resting energy expenditure. Auton Neurosci. 2012; 169:102–06. 10.1016/j.autneu.2012.05.003 22682704

[29] Shekha K, Ghosh J, Thekkoott D, Greenberg Y. Risk stratification for sudden cardiac death in patients with non-ischemic dilated cardiomyopathy. Indian Pacing Electrophysiol J. 2005; 5:122–38. 16943952 PMC1502083

[30] Malliani A, Lombardi F, Pagani M, Cerutti S. Power spectral analysis of cardiovascular variability in patients at risk for sudden cardiac death. J Cardiovasc Electrophysiol. 1994; 5:274–86. 10.1111/j.1540-8167.1994.tb01164.x 8193742

[31] Messina G, De Luca V, Viggiano A, Ascione A, Iannaccone T, Chieffi S, Monda M. Autonomic nervous system in the control of energy balance and body weight: personal contributions. Neurol Res Int. 2013; 2013:639280. 10.1155/2013/639280 23691314 PMC3649682

[32] Messina G, Chieffi S, Viggiano A, Tafuri D, Cibelli G, Valenzano A, Triggiani AI, Messina A, De Luca V, Monda M. Parachute jumping induces more sympathetic activation than cortisol secretion in first-time parachutists. Asian J Sports Med. 2016; 7:e26841. 10.5812/asjsm.26841 27217924 PMC4870822

[33] Viggiano A, Nicodemo U, Viggiano E, Messina G, Viggiano A, Monda M, De Luca B. Mastication overload causes an increase in O2- production into the subnucleus oralis of the spinal trigeminal nucleus. Neuroscience. 2010; 166:416–21. 10.1016/j.neuroscience.2009.12.071 20045451

[34] Yoshinaga M, Ushinohama H, Sato S, Tauchi N, Horigome H, Takahashi H, Sumitomo N, Kucho Y, Shiraishi H, Nomura Y, Shimizu W, Nagashima M. Electrocardiographic screening of 1-month-old infants for identifying prolonged QT intervals. Circ Arrhythm Electrophysiol. 2013; 6:932–38. 10.1161/CIRCEP.113.000619 24036083

[35] Gonzalez Corcia MC, Sieira J, Sarkozy A, de Asmundis C, Chierchia GB, Hernandez Ojeda J, Pappaert G, Brugada P. Brugada syndrome in the young: an assessment of risk factors predicting future events. Europace. 2017; 19:1864–73. 10.1093/europace/euw206 27738063

[36] Ramírez J, Orini M, Mincholé A, Monasterio V, Cygankiewicz I, Bayés de Luna A, Martínez JP, Laguna P, Pueyo E. Sudden cardiac death and pump failure death prediction in chronic heart failure by combining ECG and clinical markers in an integrated risk model. PLoS One. 2017; 12:e0186152. 10.1371/journal.pone.0186152 29020031 PMC5636125

[37] Burns KM, Bienemann L, Camperlengo L, Cottengim C, Covington TM, Dykstra H, Faulkner M, Kobau R, Erck Lambert AB, MacLeod H, Parks SE, Rosenberg E, Russell MW, et al, and Sudden Death in the Young Case Registry Steering Committee. The sudden death in the young case registry: collaborating to understand and reduce mortality. Pediatrics. 2017; 139:e20162757. 10.1542/peds.2016-2757 28228502 PMC5330401

[38] Drezner JA, Harmon KG, Asif IM, Marek JC. Why cardiovascular screening in young athletes can save lives: a critical review. Br J Sports Med. 2016; 50:1376–78. 10.1136/bjsports-2016-096606 27418320

[39] Mont L, Pelliccia A, Sharma S, Biffi A, Borjesson M, Brugada Terradellas J, Carré F, Guasch E, Heidbuchel H, La Gerche A, Lampert R, McKenna W, Papadakis M, et al, and Reviewers. Pre-participation cardiovascular evaluation for athletic participants to prevent sudden death: position paper from the EHRA and the EACPR, branches of the ESC. Endorsed by APHRS, HRS, and SOLAECE. Eur J Prev Cardiol. 2017; 24:41–69. 10.1177/2047487316676042 27815537

[40] Corrado D, Pelliccia A, Bjørnstad HH, Vanhees L, Biffi A, Borjesson M, Panhuyzen-Goedkoop N, Deligiannis A, Solberg E, Dugmore D, Mellwig KP, Assanelli D, Delise P, et al, and Study Group of Sport Cardiology of the Working Group of Cardiac Rehabilitation and Exercise Physiology and the Working Group of Myocardial and Pericardial Diseases of the European Society of Cardiology, and Consensus Statement of the Study Group of Sport Cardiology of the Working Group of Cardiac Rehabilitation and Exercise Physiology and the Working Group of Myocardial and Pericardial Diseases of the European Society of Cardiology. Cardiovascular pre-participation screening of young competitive athletes for prevention of sudden death: proposal for a common European protocol. Eur Heart J. 2005; 26:516–24. 10.1093/eurheartj/ehi108 15689345

[41] Maron BJ, Zipes DP, Kovacs RJ, and American Heart Association Electrocardiography and Arrhythmias Committee of Council on Clinical Cardiology, Council on Cardiovascular Disease in Young, Council on Cardiovascular and Stroke Nursing, Council on Functional Genomics and Translational Biology, and American College of Cardiology. Eligibility and disqualification recommendations for competitive athletes with cardiovascular abnormalities: preamble, principles, and general considerations: a scientific statement from the American Heart Association and American College of Cardiology. J Am Coll Cardiol. 2015; 66:2343–49. 10.1016/j.jacc.2015.09.032 26542655

[42] Marine JE, Shetty V, Chow GV, Wright JG, Gerstenblith G, Najjar SS, Lakatta EG, Fleg JL. Prevalence and prognostic significance of exercise-induced nonsustained ventricular tachycardia in asymptomatic volunteers: BLSA (Baltimore Longitudinal Study of Aging). J Am Coll Cardiol. 2013; 62:595–600. 10.1016/j.jacc.2013.05.026 23747767 PMC3800197

[43] Maranesi E, Morettini M, Agostinelli A, Giuliani C, Di Nardo F, Burattini L. Health monitoring in sport through wearable sensors: a novel approach based on heart-rate variability. In: Conti M., Martínez Madrid N., Seepold R., Orcioni S. (eds) Mobile Networks for Biometric Data Analysis. Lecture Notes in Electrical Engineering. Springer, Cham. 2016; 392:235–46. https://doi.org/10.1007/978-3-319-39700-9_19.

[44] Kalsbeek A, Kreier F, Fliers E, Sauerwein HP, Romijn JA, Buijs RM. Minireview: circadian control of metabolism by the suprachiasmatic nuclei. Endocrinology. 2007; 148:5635–39. 10.1210/en.2007-0776 17901232

[45] Bessem BB, de Bruijn MM, Nieuwland WW. Gender differences in the electrocardiogram screening of athletes. J Sci Med Sport. 2017; 20:213–17. 10.1016/j.jsams.2016.06.010 27451270

[46] Boos CJ, Vincent E, Mellor A, O’Hara J, Newman C, Cruttenden R, Scott P, Cooke M, Matu J, Woods DR. The effect of sex on heart rate variability at high altitude. Med Sci Sports Exerc. 2017; 49:2562–69. 10.1249/MSS.0000000000001384 28731986

[47] Mason JW, Ramseth DJ, Chanter DO, Moon TE, Goodman DB, Mendzelevski B. Electrocardiographic reference ranges derived from 79,743 ambulatory subjects. J Electrocardiol. 2007; 40:228–34. 10.1016/j.jelectrocard.2006.09.003 17276451

[48] Cygankiewicz I, Zareba W. Heart rate variability. Handb Clin Neurol. 2013; 117:379–93. 10.1016/B978-0-444-53491-0.00031-6 24095141

[49] Politano L, Palladino A, Nigro G, Scutifero M, Cozza V. Usefulness of heart rate variability as a predictor of sudden cardiac death in muscular dystrophies. Acta Myol. 2008; 27:114–22. 19472920 PMC2858940

[50] Gang Y, Malik M. Heart rate variability: measurements and risk stratification. In: Gussak I, Antzelevitch C, Wilde AAM, Friedman PA, Ackerman MJ, Shen WK (Eds). Electrical Diseases of the Heart: Genetics. Mechanisms, Treatment, Prevention. Springer, London. 2008. pp. 365–78. https://doi.org/10.1007/978-1-84628-854-8_25.

[51] Gomes ME, Aengevaeren WR, Lenders JW, Verheugt FW, Smits P, Tack CJ. Improving myocardial perfusion by percutaneous coronary intervention reduces central sympathetic activity in stable angina. Clin Cardiol. 2010; 33:E16–21. 10.1002/clc.20676 20552589 PMC6653192

[52] Gökçe M, Karahan B, Yilmaz R, Örem C, Erdöl C, Özdemir S. Long term effects of hormone replacement therapy on heart rate variability, QT interval, QT dispersion and frequencies of arrhythmia. Int J Cardiol. 2005; 99:373–79. 10.1016/j.ijcard.2003.03.030 15771916

[53] Goldkorn R, Naimushin A, Shlomo N, Dan A, Oieru D, Moalem I, Rozen E, Gur I, Levitan J, Rosenmann D, Mogilewsky Y, Klempfner R, Goldenberg I. Comparison of the usefulness of heart rate variability versus exercise stress testing for the detection of myocardial ischemia in patients without known coronary artery disease. Am J Cardiol. 2015; 115:1518–22. 10.1016/j.amjcard.2015.02.054 25872904

[54] Cornforth DJ, Jelinek HF. Detection of congestive heart failure using Renyi entropy. Comput Cardiol. 2016; 43:669–72. http://www.cinc.org/archives/2016/pdf/196-231.pdf

[55] Zöllei E, Avramov K, Gingl Z, Rudas L. Severe cardiovascular autonomic dysfunction in a patient with Guillain-Barre syndrome: a case report. Auton Neurosci. 2000; 86:94–98. 10.1016/S1566-0702(00)00188-0 11269930

[56] Videira G, Castro P, Vieira B, Filipe JP, Santos R, Azevedo E, Sá MJ, Abreu P. Autonomic dysfunction in multiple sclerosis is better detected by heart rate variability and is not correlated with central autonomic network damage. J Neurol Sci. 2016; 367:133–37. 10.1016/j.jns.2016.05.049 27423576

[57] Rodrigues D, Tran Y, Guest R, Middleton J, Craig A. Influence of neurological lesion level on heart rate variability and fatigue in adults with spinal cord injury. Spinal Cord. 2016; 54:292–97. 10.1038/sc.2015.174 26458970

[58] Romigi A, Toschi N. Cardiac autonomic changes in epilepsy. In: Barbieri R, Scilingo, EP, Valenza G (Eds). Complexity and Nonlinearity in Cardiovascular Signals. 2017; 375-386. https://doi.org/10.1007/978-3-319-58709-7_14.

[59] Ma Y, Tseng PH, Ahn A, Wu MS, Ho YL, Chen MF, Peng CK. cardiac autonomic alteration and metabolic syndrome: an ambulatory ECG-based study in a general population. Sci Rep. 2017; 7:44363. 10.1038/srep44363 28290487 PMC5349605

[60] Cugini P, Amato S, Tarquini G, Mercuri S, Turinese I, Tego A, Rossetti M, Panetti D, Filardi T, Curione M, Morano S. [Diagnosing silent cardiac dysautonomia via ambulatory blood pressure monitoring: early diagnosis shown by the lack of heart rate circadian rhythm in type 1 diabetes mellitus]. Clin Ter. 2010; 161:e1–10. 20544147

[61] Chen J, Yang SB, Liu J, Tang ZH. Diagnostic performance analysis for diabetic cardiovascular autonomic neuropathy based on short-term heart rate variability using Bayesian methods: preliminary analysis. Diabetol Metab Syndr. 2015; 7:74. 10.1186/s13098-015-0070-z 26366204 PMC4566203

[62] Kuang D, Yang R, Chen X, Lao G, Wu F, Huang X, Lv R, Zhang L, Song C, Ou S. Depression recognition according to heart rate variability using Bayesian Networks. J Psychiatr Res. 2017; 95:282–87. 10.1016/j.jpsychires.2017.09.012 28926794

[63] Meeus M, Goubert D, De Backer F, Struyf F, Hermans L, Coppieters I, De Wandele I, Da Silva H, Calders P. Heart rate variability in patients with fibromyalgia and patients with chronic fatigue syndrome: a systematic review. Semin Arthritis Rheum. 2013; 43:279–87. 10.1016/j.semarthrit.2013.03.004 23838093

[64] Yoon H, Hwang SH, Choi JW, Lee YJ, Jeong DU, Park KS. REM sleep estimation based on autonomic dynamics using R-R intervals. Physiol Meas. 2017; 38:631–51. 10.1088/1361-6579/aa63c9 28248198

[65] Lutfi MF. Autonomic modulations in patients with bronchial asthma based on short-term heart rate variability. Lung India. 2012; 29:254–58. 10.4103/0970-2113.99111 22919165 PMC3424865

[66] Chladekova L, Czippelova B, Turianikova Z, Tonhajzerova I, Calkovska A, Baumert M, Javorka M. Multiscale time irreversibility of heart rate and blood pressure variability during orthostasis. Physiol Meas. 2012; 33:1747–56. 10.1088/0967-3334/33/10/1747 23010992

[67] Mazurak N, Seredyuk N, Sauer H, Teufel M, Enck P. Heart rate variability in the irritable bowel syndrome: a review of the literature. Neurogastroenterol Motil. 2012; 24:206–16. 10.1111/j.1365-2982.2011.01866.x 22256893

[68] Hansen AL, Olson G, Dahl L, Thornton D, Grung B, Graff IE, Frøyland L, Thayer JF. Reduced anxiety in forensic inpatients after a long-term intervention with Atlantic salmon. Nutrients. 2014; 6:5405–18. 10.3390/nu6125405 25431880 PMC4276975

[69] Przybylska-Felus M, Furgala A, Zwolinska-Wcislo M, Mazur M, Widera A, Thor P, Mach T. Disturbances of autonomic nervous system activity and diminished response to stress in patients with celiac disease. J Physiol Pharmacol. 2014; 65:833–41. 25554987

[70] Beaumont A, Burton AR, Lemon J, Bennett BK, Lloyd A, Vollmer-Conna U. Reduced cardiac vagal modulation impacts on cognitive performance in chronic fatigue syndrome. PLoS One. 2012; 7:e49518. 10.1371/journal.pone.0049518 23166694 PMC3498107

[71] Elmenshawy E, Sakr S. Autonomic dysfunction in migraine; what do we need to know? Egypt J Neurol Psychiat Neurosurg. 2009; 46:489–96.

[72] Jaiswal M, McKeon K, Comment N, Henderson J, Swanson S, Plunkett C, Nelson P, Pop-Busui R. Association between impaired cardiovascular autonomic function and hypoglycemia in patients with type 1 diabetes. Diabetes Care. 2014; 37:2616–21. 10.2337/dc14-0445 24973438 PMC4140160

[73] de Andrade PE, do Amaral JA, Paiva LD, Adami F, Raimudo JZ, Valenti VE, Abreu LC, Raimundo RD. Reduction of heart rate variability in hypertensive elderly. Blood Press. 2017; 26:350–58. 10.1080/08037051.2017.1354285 28738697

[74] Baker FC, Colrain IM, Trinder J. Reduced parasympathetic activity during sleep in the symptomatic phase of severe premenstrual syndrome. J Psychosom Res. 2008; 65:13–22. 10.1016/j.jpsychores.2008.04.008 18582607 PMC2519123

[75] Liao KH, Sung CW, Chu SF, Chiu WT, Chiang YH, Hoffer B, Ou JC, Chen KY, Tsai SH, Lin CM, Chen GS, Li WJ, Wang JY. Reduced power spectra of heart rate variability are correlated with anxiety in patients with mild traumatic brain injury. Psychiatry Res. 2016; 243:349–56. 10.1016/j.psychres.2016.07.001 27449003

[76] Hon EH, Lee ST. Electronic evaluation of the fetal heart rate. viii. patterns preceding fetal death, further observations. Am J Obstet Gynecol. 1963; 87:814–26. 14085784

[77] Kleiger RE, Miller JP, Bigger JT Jr, Moss AJ. Decreased heart rate variability and its association with increased mortality after acute myocardial infarction. Am J Cardiol. 1987; 59:256–62. 10.1016/0002-9149(87)90795-8 3812275

[78] Malik M, Farrell T, Cripps T, Camm AJ. Heart rate variability in relation to prognosis after myocardial infarction: selection of optimal processing techniques. Eur Heart J. 1989; 10:1060–74. 10.1093/oxfordjournals.eurheartj.a059428 2606116

[79] Bigger JT Jr, Albrecht P, Steinman RC, Rolnitzky LM, Fleiss JL, Cohen RJ. Comparison of time- and frequency domain-based measures of cardiac parasympathetic activity in Holter recordings after myocardial infarction. Am J Cardiol. 1989; 64:536–38. 10.1016/0002-9149(89)90436-0 2773799

[80] Japundzic N, Grichois ML, Zitoun P, Laude D, Elghozi JL. Spectral analysis of blood pressure and heart rate in conscious rats: effects of autonomic blockers. J Auton Nerv Syst. 1990; 30:91–100. 10.1016/0165-1838(90)90132-3 1973426

[81] Japundzic-Zigon N. Physiological mechanisms in regulation of blood pressure fast frequency variations. Clin Exp Hypertens. 1998; 20:359–88. 10.3109/10641969809053219 9607401

[82] Nicolini P, Ciulla MM, De Asmundis C, Magrini F, Brugada P. The prognostic value of heart rate variability in the elderly, changing the perspective: from sympathovagal balance to chaos theory. Pacing Clin Electrophysiol. 2012; 35:622–38. 10.1111/j.1540-8159.2012.03335.x 22352300

[83] Fauchier L, Babuty D, Fauchier JP. Heart rate variability and prognosis in coronary artery disease. Eur Heart J. 1999; 20:1135–36. 10.1053/euhj.1999.1588 10413644

[84] Galinier M, Pathak A, Fourcade J, Androdias C, Curnier D, Varnous S, Boveda S, Massabuau P, Fauvel M, Senard JM, Bounhoure JP. Depressed low frequency power of heart rate variability as an independent predictor of sudden death in chronic heart failure. Eur Heart J. 2000; 21:475–82. 10.1053/euhj.1999.1875 10681488

[85] Huikuri HV, Stein PK. Heart rate variability in risk stratification of cardiac patients. Prog Cardiovasc Dis. 2013; 56:153–59. 10.1016/j.pcad.2013.07.003 24215747

[86] Dorrance AM, Fink G. Effects of stroke on the autonomic nervous system. Compr Physiol. 2015; 5:1241–63. 10.1002/cphy.c140016 26140717

[87] Shmuely S, van der Lende M, Lamberts RJ, Sander JW, Thijs RD. The heart of epilepsy: current views and future concepts. Seizure. 2017; 44:176–83. 10.1016/j.seizure.2016.10.001 27843098

[88] Buchheit M, Simon C, Viola AU, Doutreleau S, Piquard F, Brandenberger G. Heart rate variability in sportive elderly: relationship with daily physical activity. Med Sci Sports Exerc. 2004; 36:601–05. 10.1249/01.MSS.0000121956.76237.B5 15064587

[89] Chieffi S, Conson M, Carlomagno S. Movement velocity effects on kinaesthetic localisation of spatial positions. Exp Brain Res. 2004; 158:421–26. 10.1007/s00221-004-1916-z 15127172

[90] Chieffi S, Iavarone A, Viggiano A, Monda M, Carlomagno S. Effect of a visual distractor on line bisection. Exp Brain Res. 2012; 219:489–98. 10.1007/s00221-012-3106-8 22576681

[91] Chieffi S, Iavarone A, Iaccarino L, La Marra M, Messina G, De Luca V, Monda M. Age-related differences in distractor interference on line bisection. Exp Brain Res. 2014; 232:3659–64. 10.1007/s00221-014-4056-0 25092273

[92] Viggiano A, Chieffi S, Tafuri D, Messina G, Monda M, De Luca B. Laterality of a second player position affects lateral deviation of basketball shooting. J Sports Sci. 2014; 32:46–52. 10.1080/02640414.2013.805236 23876006

[93] Corrado D, Basso C, Rizzoli G, Schiavon M, Thiene G. Does sports activity enhance the risk of sudden death in adolescents and young adults? J Am Coll Cardiol. 2003; 42:1959–63. 10.1016/j.jacc.2003.03.002 14662259

[94] Harmon KG, Drezner JA, Wilson MG, Sharma S. Incidence of sudden cardiac death in athletes: a state-of-the-art review. Heart. 2014; 100:1227–34. 10.1136/heartjnl-2014-093872.rep 25049314

[95] Schmied C, Borjesson M. Sudden cardiac death in athletes. J Intern Med. 2014; 275:93–103. 10.1111/joim.12184 24350833

[96] Cipryan L, Tschakert G, Hofmann P. Acute and post-exercise physiological responses to high-intensity interval training in endurance and sprint athletes. J Sports Sci Med. 2017; 16:219–29. 28630575 PMC5465984

[97] Triggiani AI, Valenzano A, Ciliberti MA, Moscatelli F, Villani S, Monda M, Messina G, Federici A, Babiloni C, Cibelli G. Heart rate variability is reduced in underweight and overweight healthy adult women. Clin Physiol Funct Imaging. 2017; 37:162–67. 10.1111/cpf.12281 26211739

[98] Valenzano A, Moscatelli F, Triggiani AI, Capranica L, De Ioannon G, Piacentini MF, Mignardi S, Messina G, Villani S, Cibelli G. Heart-rate changes after an ultraendurance swim from Italy to Albania: A case report. Int J Sports Physiol Perform. 2016; 11:407–09. 10.1123/ijspp.2015-0035 26263484

[99] Maron BJ, Pelliccia A. The heart of trained athletes: cardiac remodeling and the risks of sports, including sudden death. Circulation. 2006; 114:1633–44. 10.1161/CIRCULATIONAHA.106.613562 17030703

[100] Nolan JP, Soar J, Zideman DA, Biarent D, Bossaert LL, Deakin C, Koster RW, Wyllie J, Böttiger B, and ERC Guidelines Writing Group. European Resuscitation Council Guidelines for Resuscitation 2010 Section 1. Executive summary. Resuscitation. 2010; 81:1219–76. 10.1016/j.resuscitation.2010.08.021 20956052

[101] Borjesson M, Serratosa L, Carre F, Corrado D, Drezner J, Dugmore DL, Heidbuchel HH, Mellwig KP, Panhuyzen-Goedkoop NM, Papadakis M, Rasmusen H, Sharma S, Solberg EE, et al, and writing group on behalf of the EACPR Section of Sports Cardiology. Consensus document regarding cardiovascular safety at sports arenas: position stand from the European Association of Cardiovascular Prevention and Rehabilitation (EACPR), section of Sports Cardiology. Eur Heart J. 2011; 32:2119–24. 10.1093/eurheartj/ehr178 21672932

[102] La Rovere MT, Pinna GD, Maestri R, Mortara A, Capomolla S, Febo O, Ferrari R, Franchini M, Gnemmi M, Opasich C, Riccardi PG, Traversi E, Cobelli F. Short-term heart rate variability strongly predicts sudden cardiac death in chronic heart failure patients. Circulation. 2003; 107:565–70. 10.1161/01.CIR.0000047275.25795.17 12566367

[103] Terziyski KV, Draganova AI, Taralov ZZ, Ilchev IS, Kostianev SS. The effect of continuous positive airway pressure on heart rate variability during the night in patients with chronic heart failure and central sleep apnoea. Clin Exp Pharmacol Physiol. 2016; 43:1185–90. 10.1111/1440-1681.12662 27560005

[104] Yang Y, Aro AL, Nair SG, Jayaraman R, Reinier K, Rusinaru C, Uy-Evanado A, Yarmohammadi H, Jui J, Chugh SS. Novel measure of autonomic remodeling associated with sudden cardiac arrest in diabetes. Heart Rhythm. 2017; 14:1449–55. 10.1016/j.hrthm.2017.07.011 28711633 PMC5624843

[105] Kiviniemi AM, Tulppo MP, Wichterle D, Hautala AJ, Tiinanen S, Seppänen T, Mäkikallio TH, Huikuri HV. Novel spectral indexes of heart rate variability as predictors of sudden and non-sudden cardiac death after an acute myocardial infarction. Ann Med. 2007; 39:54–62. 10.1080/07853890600990375 17364451

[106] Huang WA, Boyle NG, Vaseghi M. Cardiac innervation and the autonomic nervous system in sudden cardiac death. Card Electrophysiol Clin. 2017; 9:665–79. 10.1016/j.ccep.2017.08.002 29173409 PMC5777242

[107] Oliva A, Grassi VM, Campuzano O, Brion M, Arena V, Partemi S, Coll M, Pascali VL, Brugada J, Carracedo A, Brugada R. Medico-legal perspectives on sudden cardiac death in young athletes. Int J Legal Med. 2017; 131:393–409. 10.1007/s00414-016-1452-y 27654714

